# Hirsutanol A Attenuates Lipopolysaccharide-Mediated Matrix Metalloproteinase 9 Expression and Cytokines Production and Improves Endotoxemia-Induced Acute Sickness Behavior and Acute Lung Injury

**DOI:** 10.3390/md17060360

**Published:** 2019-06-17

**Authors:** Jing-Shiun Jan, Chih-Hao Yang, Mong-Heng Wang, Fan-Li Lin, Jing-Lun Yen, Irene Hsieh, Maksim Khotimchenko, Tzong-Huei Lee, George Hsiao

**Affiliations:** 1 Graduate Institute of Medical Sciences, College of Medicine, Taipei Medical University, Taipei 110, Taiwan; d119101004@tmu.edu.tw (J.-S.J.); chyang@tmu.edu.tw (C.-H.Y.); fllin@tmu.edu.tw (F.-L.L.); m120102039@tmu.edu.tw (J.-L.Y.); m120105021@tmu.edu.tw (I.H.); 2 Department of Pharmacology, School of Medicine, College of Medicine, Taipei Medical University, Taipei 110, Taiwan; 3 Department of Physiology, Augusta University, Augusta, GA 30912, USA; mwang@augusta.edu; 4 School of Biomedicine, Far Eastern Federal University, 690091 Vladivostok, Russia; khotimchenko.my@dvfu.ru; 5 Institute of Fisheries Science, National Taiwan University, Taipei 106, Taiwan; thlee1@ntu.edu.tw; 6 Ph.D. Program in Biotechnology Research and Development, College of Pharmacy, Taipei Medical University, Taipei 110, Taiwan

**Keywords:** signal transducer and activator of transcription 3 (STAT3), matrix metalloproteinases-9 (MMP-9), interleukin (IL), lipopolysaccharide (LPS), acute sickness behavior, acute lung injury (ALI)

## Abstract

Activated human monocytes/macrophages, which increase the levels of matrix metalloproteinases (MMPs) and pro-inflammatory cytokines, are the essential mechanisms for the progression of sepsis. In the present study, we determined the functions and mechanisms of hirsutanolA (HA), which is isolated from the red alga-derived marine fungus *Chondrostereum* sp. NTOU4196, on the production of pro-inflammatory mediators produced from lipopolysaccharide (LPS)-treated THP-1 cells. Our results showed that HA suppressed LPS-triggered MMP-9-mediated gelatinolysis and expression of protein and mRNA in a concentration-dependent manner without effects on TIMP-1 activity. Also, HA significantly attenuated the levels of TNF-α, IL-6, and IL-1β from LPS-treated THP-1 cells. Moreover, HA significantly inhibited LPS-mediated STAT3 (Tyr705) phosphorylation, IκBα degradation and ERK1/2 activation in THP-1 cells. In an LPS-induced endotoxemia mouse model, studies indicated that HA pretreatment improved endotoxemia-induced acute sickness behavior, including acute motor deficits and anxiety-like behavior. HA also attenuated LPS-induced phospho-STAT3 and pro-MMP-9 activity in the hippocampus. Notably, HA reduced pathologic lung injury features, including interstitial tissue edema, infiltration of inflammatory cells and alveolar collapse. Likewise, HA suppressed the induction of phospho-STAT3 and pro-MMP-9 in lung tissues. In conclusion, our results provide pharmacological evidence that HA could be a useful agent for treating inflammatory diseases, including sepsis.

## 1. Introduction

Sepsis is defined as an abnormal host response to infection, which triggers inflammation throughout the body and causes life-threatening organ dysfunction [1]. Sepsis and septic shock are significant public health concerns, affecting millions of people around the world with high mortality and poor diagnosis [1,2,3]. Notably, in-hospital mortality of sepsis has declined in the last two decades because of established evidence-based managements, which improve the outcome of the critically ill patient [2,4,5]. Nevertheless, deterioration of health after sepsis is still common, including physical disability and cognition impairment [3]. Because of the high mortality rate and serious complications, it is critical to develop new and novel approaches to the treatment of sepsis.

The innate immune system is the first line of defense against microorganism, but the uncontrolled inflammatory response can lead to tissue damage [6,7]. Tissue-resident leukocytes recognize the molecules released from pathogens and dead host cells via a pattern recognition receptor activating downstream transcription factors, especially NF-κB and AP-1, leading to the production of inflammatory mediators. These inflammatory mediators then initiate localized transmigration of leukocytes to the infection site to destroy pathogens [7]. The positive feedback loop between cytokines and immune cells leads to an auto-amplifying phenomenon and cytokine storm, eventually resulting in injuries to multiple organs during sepsis [7,8,9]. The emblematic pro-inflammatory cytokines, including tumor necrosis factor (TNF)-α, interleukin (IL)-1β and IL-6 are major products generated by monocyte/macrophages and neutrophils. These pro-inflammatory cytokines play a critical role in pathophysiologic processes of sepsis associated-organ injuries and -cognition impairment, as described previously [9,10]. Therefore, pro-inflammatory cytokines are considered as the therapeutic targets for the treatment of sepsis or as diagnostic biomarkers of sepsis [11,12].

Matrix metalloproteinases (MMPs) are zinc- and calcium-dependent endopeptidases; these enzymes involve in biological processes including tissue remodeling and angiogenesis via degrading the extracellular matrix [13]. Also, MMPs can regulate non-matrix substrates in pathological processes. For example, MMP-9 has been shown to cleave and activate pro-inflammatory cytokines, and degrade the complement of tight junction protein [14]. Therefore, several reports [13,15,16,17] have shown that MMP-9 plays an essential role in the pathogenesis of inflammation diseases, such as multiple sclerosis, arthritis, ischemic stroke, and sepsis. Leukocytes-mediated MMP-9 production and secretion could be induced by inflammatory response, in particular endotoxemia, CD11b^high^ GR-1^high^ neutrophils are the main producers of MMP-9 [18,19]. The serum levels of MMP-9 are elevated in patients with both sepsis and septic animal models [13,20]. The MMP-deficient mice have low lethality in a lipopolysaccharide (LPS)-induced endotoxemia model [21]. In addition, synthetic inhibitor Regasepin1-inhibiting neutrophil MMP-9 and MMP-8 protects mice against endotoxin shock [22]. Further, MMP-9 blockade attenuated blood-brain-barrier dysfunction in a cecal ligation and puncture rat model of sepsis [23]. Thus, MMP-9 inhibition could provide a therapeutic strategy of sepsis.

Hirsutanol A (HA), a hirsutane-type sesquiterpene, which is isolated from marine fungus *Chondrostereum* sp. NTOU4196, attenuates NO production in LPS-activated murine BV-2 microglial cells as described previously [24]. This report [24] supports the notion that HA could be a potential anti-inflammatory agent. In this study, we determined the inhibitory effect and mechanism of HA on production of MMP-9 and pro-inflammatory cytokine (TNF-α, IL-1β, and IL-6) from LPS-treated human monocytic THP-1 cells. In an LPS-induced endotoxemia mouse model, we further evaluated the protective effect of HA on endotoxemia-induced acute sickness behavior and acute lung injury (ALI).

## 2. Results

### 2.1. HA Inhibited MMP-9-Mediated Gelatinolysis Induced by LPS

**Figure 1:**
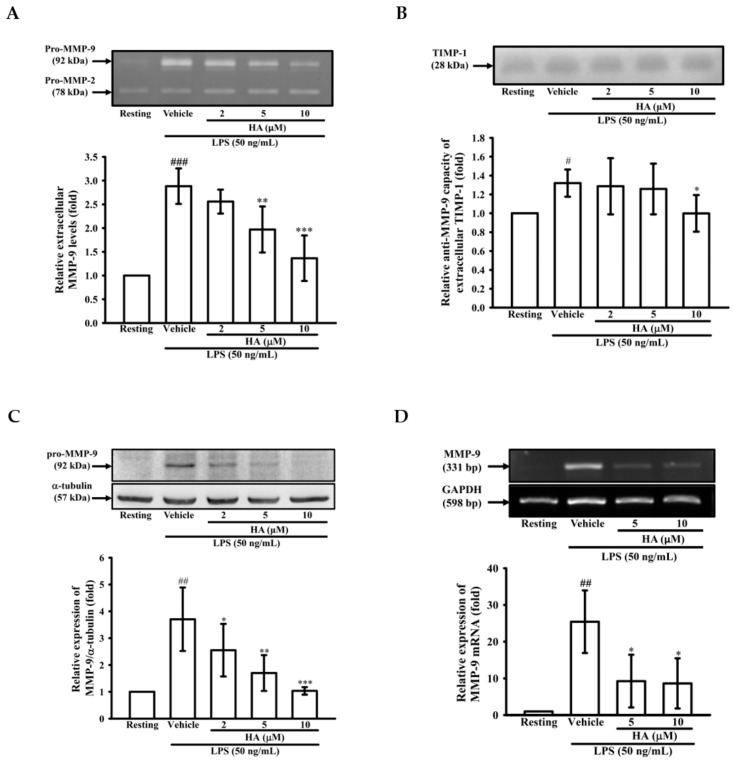
Effect of HA on MMP-9-mediated gelatinolysis and expression induced by LPS. THP-1 cells (5 × 10^5^ cells/0.5 mL) were dispensed onto 24-well plates and treated with LPS (50 ng/mL) for 24 h. Cells were treated with the indicated concentrations of HA (2, 5 and 10 μM) or vehicle for 15 min before treatment with a stimulant. Cell-free supernatants were then assayed for MMPs and TIMP-1 activity by gelatin zymography (**A**) and reverse zymography (**B**). THP-1 cells (10^6^ cells/mL) were dispensed onto 6-well plates and were treated with LPS (50 ng/mL) for 24 h (**C**) or 8 h (**D**) at the indicated concentrations of HA or vehicle for 15 min before treatment with LPS. Cell lysates were obtained and analyzed for MMP-9 protein expression by Western blotting or for MMP-9 mRNA expression by RT-PCR. Data represent means ± S.D. from three independent experiments. ^#^ *p* < 0.05, ^##^ *p* < 0.01 and ^###^ *p* < 0.001 as compared with the resting, * *p* < 0.05, ** *p* < 0.01 and *** *p* < 0.001 as compared with the vehicle.

To determine whether HA modulated the production of MMP-9, MMP-2, or TIMP-1 from LPS-activated monocytes, THP-1 cells were pretreated with HA (2, 5, and 10 μM) for 15 min, followed by the addition of LPS (50 ng/mL) within 24 h. Zymography showed that LPS (50 ng/mL) significantly enhanced extracellular MMP-9-mediated gelatinolysis by up to 3.06 ± 0.12-fold, and pretreatment with HA (2, 5, and 10 μM) strongly inhibited MMP-9-mediated gelatinolysis in a concentration-dependent manner by 2.88 ± 0.37-, 2.56 ± 0.25-, 1.97 ± 0.49-, and 1.37 ± 0.48-fold compared with that under the normal condition, respectively (Figure 1A). In addition, the pretreatment of HA (10 μM) had no effect on monocytic extracellular MMP-9 gelatinolysis in the absence of LPS (Figure S1). Systematic TIMP-1 protein levels are increased during endotoxemia [18]. Similarly, as shown in Figure 1B, THP-1 cells constitutively releasing TIMP-1 was enhanced by LPS stimulation. The pretreatment of HA did not upregulate the TIMP-1 production of THP-1. In contrast, HA (10 μM) attenuated TIMP-1 enhanced by LPS challenge. Furthermore, to confirm whether HA suppressed extracellular MMP-9 gelatinolysis by down-regulating MMP-9 expression, intracellular MMP-9 protein expression was evaluated by immunoblotting. As shown in Figure 1C, compared with the resting condition, THP-1 cells treated with LPS for 24 h strongly induced up-regulation of MMP-9 protein expression by up to 3.71 ± 1.18-fold compared with that under the normal condition. Pretreatment with HA at different concentrations for 15 min followed by the addition of LPS (50 ng/mL) within 24 h decreased MMP-9 expression to 2.55 ± 0.98-, 1.70 ± 0.67-, and 1.04 ± 0.14-fold, respectively, in a concentration-dependent manner. Similarly, LPS (50 ng/mL) significantly increased the expression of MMP-9 mRNA in THP-1 cells by up to 25.42 ± 8.53-fold compared with normal conditions, and pretreated with HA (5 and 10 μM) significantly suppressed LPS-mediated MMP-9 mRNA expression down to 9.26 ± 7.17- and 8.62 ± 6.84-fold, respectively (Figure 1D). These results suggested that HA down-regulated MMP-9-mediated gelatinolysis occurred at both transcriptional and protein expression levels.

### 2.2. HA Attenuated Pro-Inflammatory Cytokines Production Induced by LPS

**Figure 2:**
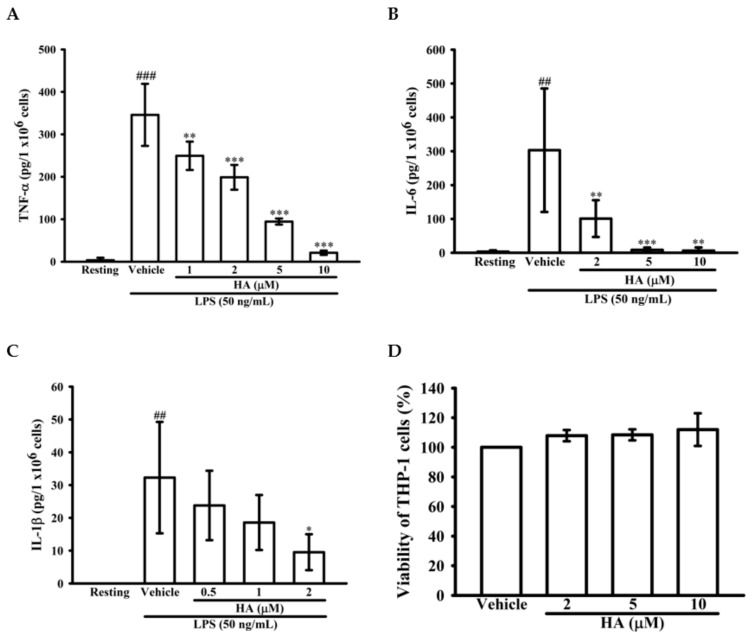
Effect of HA on pro-inflammatory cytokines production induced by LPS in THP-1 cells without cellular toxicity. THP-1 cells (5 × 10^5^ cells/0.5 mL) were dispensed onto 24-well plates and were treated with the indicated concentrations of HA or vehicle for 15 min followed by treatment with LPS (50 ng/mL) for 4 h (**A**) and 24 h (**B**,**C**). Cell-free supernatants were then assayed for the level of TNF-α (**A**), IL-6 (**B**) and IL-1β (**C**) by ELISA. (**D**) THP-1 cells treated with the indicated concentrations of HA or vehicle for 24 h. Cell viability was quantified by the ability of mitochondria to reduce the tetrazolium dye MTT in viable cells. Data represent means ± S.D. from three independent experiments. ^##^ *p* < 0.01 and ^###^ *p* < 0.001 as compared with the resting; * *p* < 0.05, ** *p* < 0.01 and *** *p* < 0.001 as compared with the vehicle.

To determine whether HA down-regulated the level of the pro-inflammatory cytokines TNF-α, IL-6, and IL-1β production by activated monocytes, THP-1 cells were pretreated with different concentrations of HA (0.5–10 μM) for 15 min followed by the addition of LPS (50 ng/mL) within 4 h or 24 h. As shown in Figure 2A, compared with the resting condition, THP-1 cells stimulated with LPS for 4 h significantly increased extracellular TNF-α levels by up to 345.8 ± 73.0 pg/10^6^ cells, and pretreatment with HA at different concentrations significantly attenuated the extracellular TNF-α production in a concentration-dependent manner to 249.5 ± 33.5, 198.8 ± 29.1, 94.5 ± 7.1, and 21.0 ± 5.1 pg/10^6^ cells, respectively. Likewise, LPS significantly increased extracellular IL-6 levels by up to 303.0 ± 182.5 pg/10^6^ cells. Pretreatment with different concentrations of HA significantly suppressed the extracellular IL-6 levels in a concentration-dependent manner to 101.0 ± 54.2, 8.5 ± 6.8, and 6.2 ± 9.5 pg/10^6^ cells, respectively (Figure 2B). Also, as shown in Figure 2C, LPS markedly enhancing extracellular IL-1β production to 32.3 ± 17.0 pg/10^6^ cells, and pretreatment with HA at different concentrations significantly decreased the extracellular IL-1β levels in a concentration-dependent manner to 23.8 ± 10.6, 18.6 ± 8.4, and 9.5 ± 5.5 pg/10^6^ cells, respectively (Figure 2C). In addition, the pretreatment of HA (5 μM) did not induce pro-inflammatory cytokines production of THP-1 without LPS challenge (Figure S2). Notably, HA (2, 5, and 10 μM) and the combination of LPS and HA (10 μM) did not have a significant cytotoxic effect against THP-1 cells based on MTT assay, respectively (Figure 2D and Figure S3).

### 2.3. HA Suppressed IκB-α Degradation and the Phosphorylation Level of STAT3 and ERK1/2 Induced by LPS

**Figure 3:**
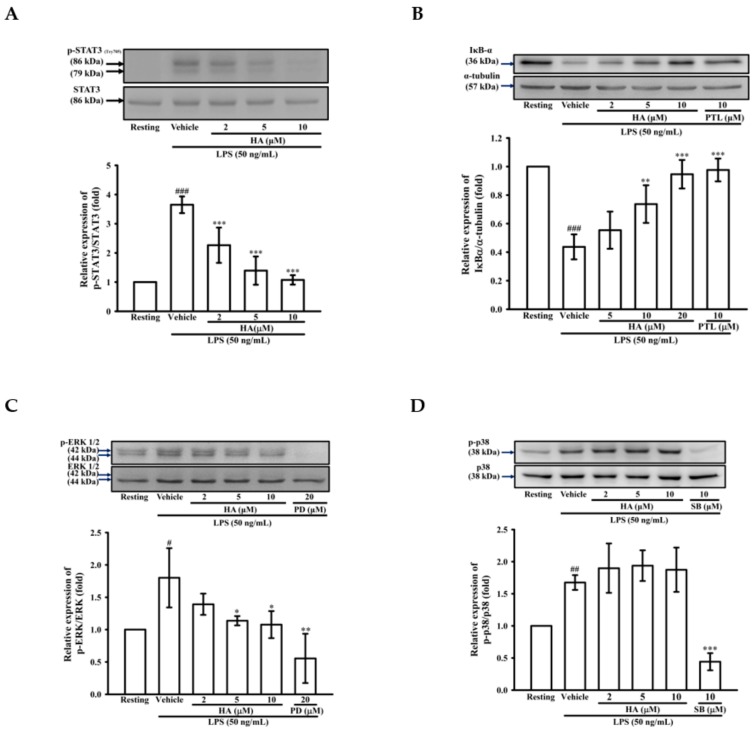
Effect of HA on STAT3, MAPK and NF-κB activation induced by LPS in THP-1 cells. THP-1 cells (10^6^ cells/mL) were dispensed onto 6-well plates and were treated with the indicated concentrations of HA (2, 5 and 10 μM), parthenolide (PTL, 10 μM), PD98059 (PD, 20 μM), SB203580 (SB, 10 μM) or vehicle followed by treatment of LPS (50 ng/mL) for 24 h (**A**), 60 min (**B**), and 30 min (**C**,**D**), respectively. Cell lysates were obtained and analyzed for IκBα degradation and the level of phosphorylated-STAT3, ERK and p38 MAPK by Western blotting. Data represent means ± S.D. from three independent experiments. ^#^ *p* < 0.05, ^##^ *p* < 0.01 and ^###^ *p* < 0.001 as compared with the resting; * *p* < 0.05, ** *p* < 0.01 and *** *p* < 0.001 as compared with the vehicle.

While the JAK2-STAT3 pathway is essential to regulate the immune response, it is considered to be involved in an “early” inflammatory response during sepsis [25]. To examine the effects of HA on LPS-mediated STAT3 activation, the phosphorylation levels of STAT3 (Tyr705) were determined by western blotting. THP-1 cells stimulated with LPS for 24 h significantly induced the level of phosphorylated-STAT3 (Tyr705) by up to 3.65 ± 0.29-fold, and pretreatment of HA (2, 5, and 10 μM) significantly attenuated LPS-mediated activation of STAT3 (Tyr705) down to 2.27 ± 0.60-, 1.39 ± 0.48-, and 1.08 ± 0.16-fold, respectively (Figure 3A). On the other hand, MMP-9 expression was regulated by various transcription factors, such as NF-κB, AP-1, and SP-1 [26]. Therefore, the effect of HA on the LPS-induced activation of MAPKs (p38, ERK), degradation of the inhibitor of kappa B alpha (IκBα) and the phosphorylation level of IκB kinase alpha/beta (IKKα/β) was determined by western blotting. To examine whether HA regulates the IKKα/IκBα/NF-κB cascade, THP-1 cells were pretreated with HA (2, 5, and 10 μM) or parthenolide (PTL; 10μM, an inhibitor of the NF-κB pathway) for 15 min followed by the addition of LPS (50 ng/mL) within 60 min. As shown in Figure 3B, significant degradation of IκBα was observed in THP-1 stimulated with LPS (50 ng/mL) for 60 min, and the pretreatment with different concentrations of HA significantly prevented the degradation of IκBα in a concentration-dependent manner by 0.55 ± 0.13-, 0.74 ± 0.13-, and 0.95 ± 0.10-fold, respectively. Similarly, LPS significantly enhanced the level of phosphorylated IKKα by up to 4.60 ± 0.72-fold, and pretreatment with HA (5 and 10 μM) attenuated the IKKα phosphorylation down to 3.79 ± 0.27-, and 3.14 ± 0.16-foldcompared with basal levels of the normal condition, respectively (Figure S4). To investigate whether HA regulates the activation of ERK or p38 MAPK, THP-1 cells were pretreated with HA (2, 5, and 10 μM), PD (PD98059, a MEK MAPK inhibitor; 20 μM), or SB (SB203580, a p38 MAPK inhibitor; 10 μM) for 15 min followed by the stimulation of LPS within 30 min. As shown in Figure 3C, the phosphorylation levels of ERK was strongly induced by LPS to 1.80 ± 0.46-fold compared with the resting condition, while the pretreatment with HA at different concentrations significantly inhibited LPS-stimulated ERK phosphorylation in a concentration-dependent manner by 1.39 ± 0.16-, 1.14 ± 0.03-, and 1.08 ± 0.21-fold, respectively. Notably, LPS significantly enhanced the phosphorylation levels of p38 by up to 1.68 ± 0.12-fold, and pretreatment with SB (10 μM) attenuated the p38 phosphorylation down to 0.44 ± 0.13-fold compared with the resting condition. However, unlike SB, pretreatment with HA at different concentrations did not affect p38 activation (Figure 3D). On the other hand, the pretreatment of HA (10 μM) did not induce phosphorylation of STAT3 and degradation of IκBα in THP-1 without LPS challenge (Figure S5). These results suggested that HA attenuated MMP-9 expression through regulating IKKα/IκBα/NF-κB- and ERK-associated signaling in THP-1 cells during LPS treatment.

### 2.4. The Activation of JAK2-STAT3 Cascade Was Associated with LPS-Mediated MMP-9 Production

**Figure 4:**
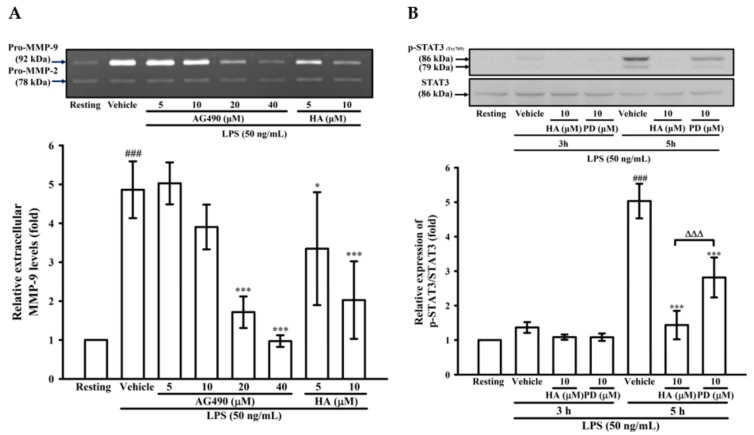

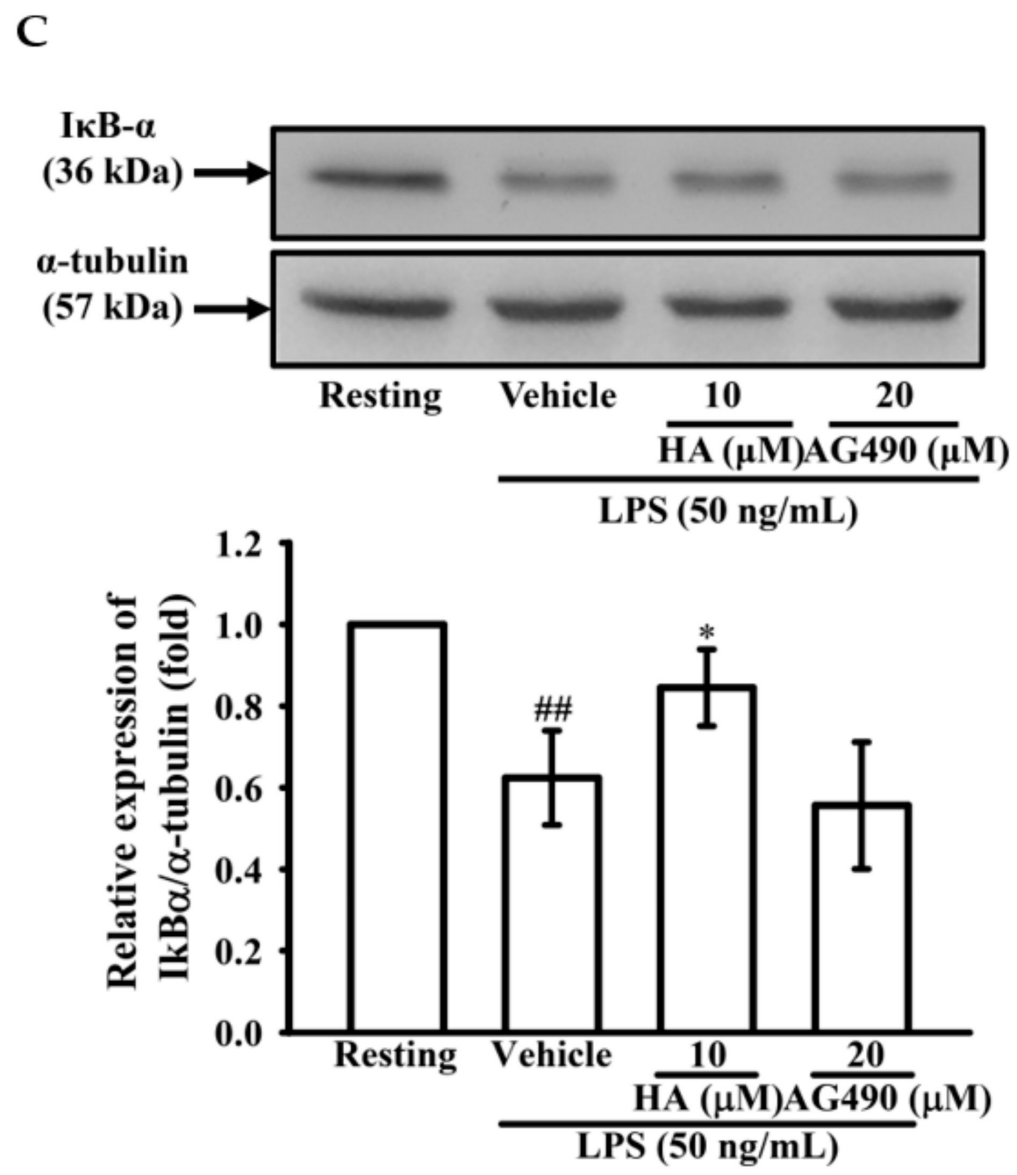
Effect of JAK2-STAT3 cascade inhibition on LPS-mediated MMP-9 expression. THP-1 cells (5 × 10^5^ cells/0.5 mL) were dispensed onto 24-well plates and treated with LPS (50 ng/mL) for 24 h. Cells were treated with the indicated concentrations of AG490 (5, 10, 20, and 40 μM), HA (5 and 10 μM) or vehicle for 15 min before treatment with LPS. Cell-free supernatants were then assayed for MMPs activity by gelatin zymography (**A**). THP-1 cells (10^6^ cells/mL) were dispensed onto 6-well plates and were treated with LPS (50 ng/mL) for 3 h, 5h (**B**) and 60 min (**C**) at the indicated concentrations of HA, PD (10 μM), AG490 (10 μM) or vehicle for 15 min before treatment with LPS. Cell lysates were obtained and analyzed for STAT3 (Tyr705) phosphorylation and IκBα degradation by Western blotting. Data represent means ± S.D. from three to four independent experiments, respectively. ^##^ *p* < 0.01 and ^###^ *p* < 0.001 as compared with the resting; * *p* < 0.05 and *** *p* < 0.001 as compared with the vehicle; ^∆∆∆^ *p* < 0.001 as compared with the group pretreated with HA pre-treatment.

To determine the effects of JAK2-STAT3 inhibition on MMP-9 production, THP-1 cells were pretreated with AG490 (a JAK2 inhibitor; 5, 10, 20, and 40 μM) or HA (5, and 10μM) for 15 min followed by the addition of LPS (50 ng/mL) for 24 h. As described in zymography, compared with the LPS-stimulating condition, the pretreatment of AG490 (20, 40 μM) significantly lessened extracellular MMP-9-mediated gelatinolysis down to 1.71 ± 0.41-, and 0.92 ± 0.15-fold, respectively (Figure 4A). Interestingly, at the same concentration, HA was more potent than AG490 on attenuating MMP-9-mediated gelatinolysis induced by LPS. The ERK1/2 inhibition suppress LPS-mediated STAT3 (Tyr705) activation and AP-1 expression in THP-1 cells [27]. Also, the inhibition of JAK2-STAT3 cascade lessens NF-κB phosphorylation in LPS-stimulated THP-1 cells [25]. As shown in Figure 3, HA pretreatment both down-regulated ERK1/2 phosphorylation and prevented IκBα degradation in LPS-challenged THP-1 cells. To investigate the difference between MEK inhibition and HA pretreatment on LPS-mediated STA3 (Tyr705) phosphorylation and whether HA regulated STAT3 activation in LPS-challenged-monocytes, THP-1 cells were pretreated with HA (10 μM) or PD (10 μM) for 15 min followed by the addition of LPS within 3 h or 5 h. As shown in Figure 4B, THP-1 cells stimulated with LPS for 5 h significantly induced STAT3 (Tyr 705) phosphorylation by up to 5.03 ± 0.50-fold, and pretreatment with HA (10 μM) significantly inhibited the phosphorylation levels to 1.44 ± 0.41-fold (Figure 4B). At the same concentration, HA was more potent than PD to inhibit STAT3 phosphorylation. To compare the difference between the effect of HA and JAK2-inhibitor on LPS-mediated IκBα/NF-κB cascade, THP-1 cells were pretreated with HA (10 μM) or AG490 (20 μM) for 15 min followed by the addition of LPS for 60 min. As indicated in Figure 4C, HA (10 μM) and AG490 (20 μM) caused similar efficacy on the inhibition of extracellular MMP-9 activity. At the same condition as stated above, HA was more effective than JAK inhibitor for preventing LPS-induced IκBα degradation in THP-1 cells. Taken together, these results suggested that HA regulated the functions of various signal molecules, including ERK1/2, NF-κB, and STAT3 to regulate the production of MMP-9 and pro-inflammatory cytokines.

### 2.5. HA Decreased LPS-Mediated Acute Sickness Behavior

**Figure 5:**
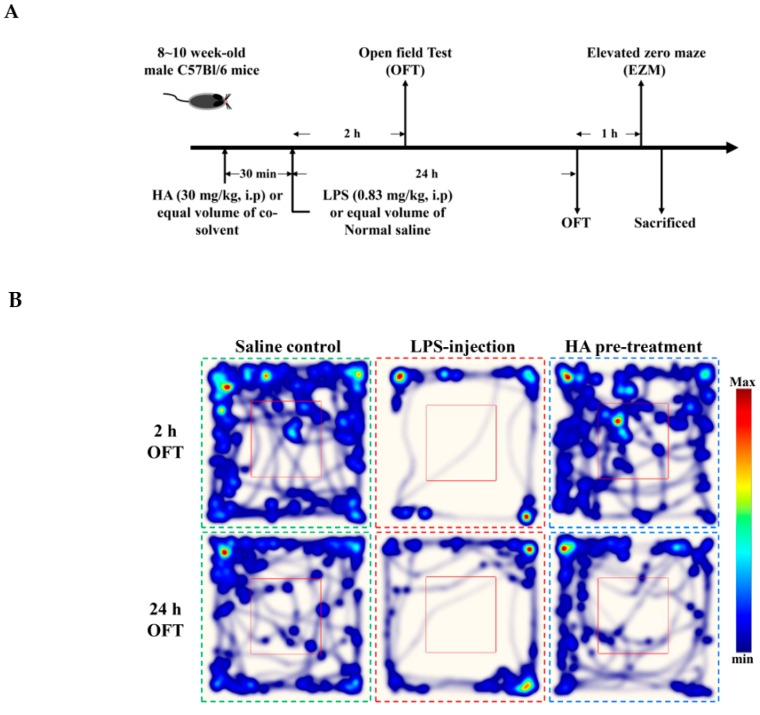

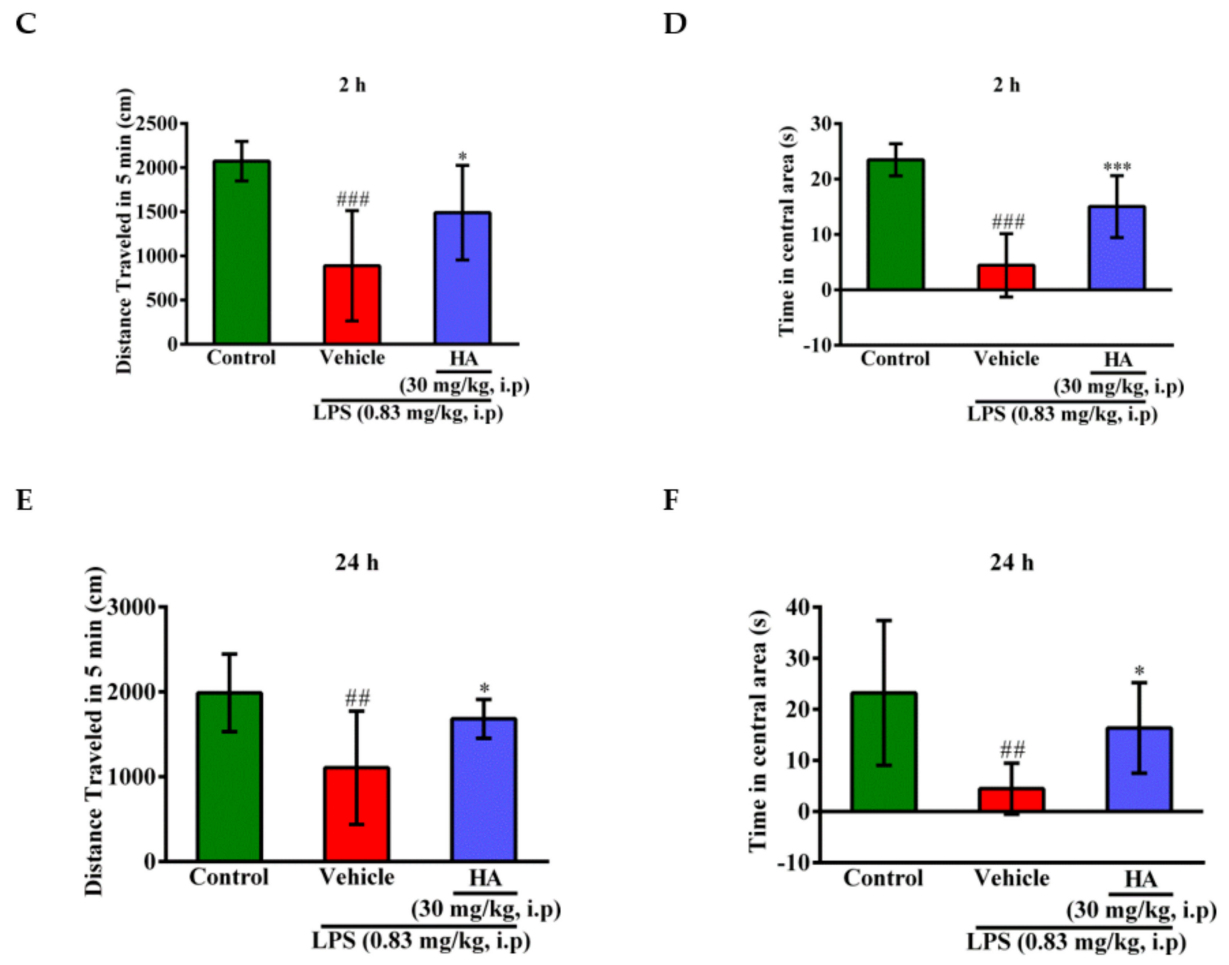
Effect of HA on sickness behavior in the open field test following a systemic LPS challenge. (**A**) Experimental timeline and design. (**B**) Heat-map represented the tracks of mice in the different groups in OFT, red = more time, blue = less time. (**C**) Total distance traveled 5 min in the different groups administrated with sterile saline or LPS (0.83 mg/kg, i.p) for 2 h. (**D**) Percentage of time spent in 5 min in the open area in the different groups for 2 h. (**E**) Total distance traveled 5 min in the different groups challenged with sterile saline or LPS (0.83 mg/kg, i.p) for 24 h. (**F**) Percentage of time spent in 5 min in the central area in the different groups for 24 h. Data represent group means ± S.D., *n* = 7 mice/group. ^##^ *p* < 0.01 and ^###^ *p* < 0.001 as compared with the saline control group (Control); * *p* < 0.05 and *** *p* < 0.001 as compared with the co-solvent group (vehicle).

**Figure 6:**
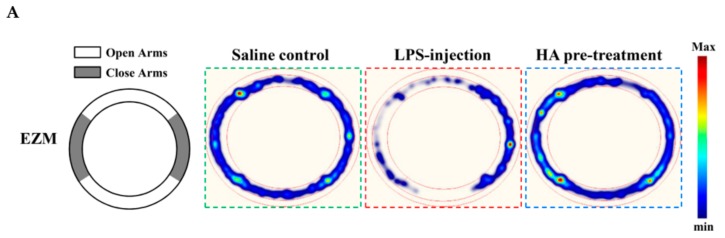

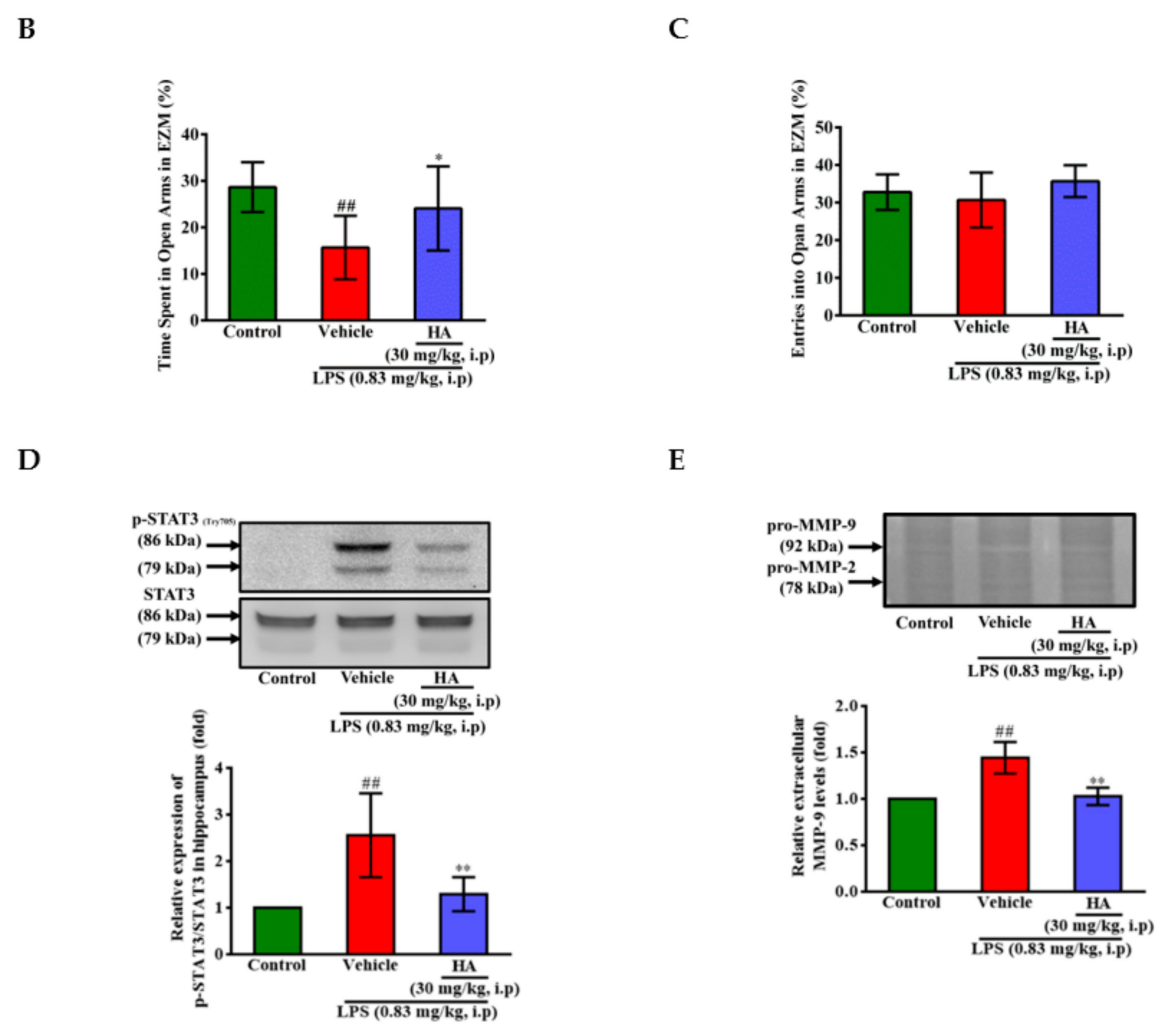
Effect of HA on anxiety-like behavior in the elevated zero maze test, and the expression of phospho-STAT3 and MMP-9 in the hippocampus following systemic LPS challenge. (**A**) The structure diagram of EZM was described as the left panel. Heat-map illustrated the tracks of mice in the different groups in EZM test, red = more time, blue = less time. (**B**) Percentage of time spent in 5 min in the open arms (gray) in the different groups administrated with sterile saline or LPS (0.83 mg/kg, i.p) for 25 h. (**C**) Percentage of entries into the open arms in 5 min in the different groups for 25 h. Mice were sacrificed after behavior tests, and then hippocampus was collected immediately. Homogenates of the hippocampus were obtained and analyzed for the phosphorylation of STAT3 by Western blotting (**D**, *n* = 5), and assayed for MMPs activity by gelatin zymography (**E**, *n* = 3). Data represent group means ± S.D., *n* = 3 to 7 mice/group. ^##^ *p* < 0.01 as compared with the saline control group (Control); * *p* < 0.05 and ** *p* < 0.01 as compared with the co-solvent group (vehicle).

Body weight loss, acute motor deficits, and anxiety-like behavior are typical sickness behaviors that are mediated by systemic immune responses [28]. As indicated in Figure 5A, the 5-min period of OFT after LPS-injection for 2 h and 24 h, respectively, were presented using heat-maps. The total distance traveled by LPS-challenged mice was dramatically reduced by 57% compared to control mice (Saline-2073.0 ± 224.9 vs. LPS-888.9 ± 625.5, *p* < 0.001). HA (30 mg/kg) pretreatment significantly prevented LPS-induced acute locomotor deficits compared to the LPS-challenged group at 2 h (Figure 5C, *p* < 0.05). As shown in Figure 5D, the LPS-injected mice group spent significantly less time in the open area than the control group (*p* < 0.001). HA pretreatment significantly lessened the LPS-induced reduction in the time spent in the open area (*p* < 0.001). One day after LPS injection, HA pretreatment still significantly improved the reduction of the time spent in the open area and the total distance traveled induced by LPS-injection (*p* < 0.05, Figure 5E,F). Also, pretreatment reduced body weight loss and body temperature abnormalities mediated by LPS at 24 h compared with the LPS-challenged group (Figure S6A,B). As shown in Figure 6 A, the 5-min period of the EZM tests were tracked as a Heat-map at one day after the LPS injection. As shown in Figure 6B, the percentage of entries in the open quadrants was significantly lower in the LPS-challenged group than in the control group (*p* < 0.01). HA-pretreatment significantly lessened the decline of time spent in the open quadrants compared to the behaviors observed in the LPS challenge group (*p* < 0.05). A slight decrease in the percentage of entries in the open quadrants was observed in the LPS challenge group compared to the control group, but no significant differences were observed among the 3 groups (*p* > 0.05, Figure 6C). To further determine whether HA pretreatment regulated STAT3 activation and pro-MMP-9 expression in the hippocampus during endotoxemia-mediated acute sickness behavior and anxiety-like behavior, the expression levels of phosphorylated STAT3 (Tyr705) and pro-MMP-9 expression were evaluated by immunoblotting and gelatin zymography, respectively. Compared with the control group, LPS significantly increased the expression levels of STAT3 phosphorylated on Tyr705 in the hippocampus to 2.55 ± 0.90 fold. The increase in phosphorylated STAT3 protein levels was reduced by HA pretreatment to 50% as compared with the LPS-challenged group (LPS-2.55 ± 0.90-fold vs. HA-1.29 ± 0.37 fold, *p* < 0.01) (Figure 6D). Additionally, zymography showed that sample of hippocampus post-LPS challenged significantly enhanced MMP-9-mediated gelatinolysis by up to 1.44 ± 0.17-fold, and pretreatment with HA significantly attenuated MMP-9-mediated gelatinolysis by 28% as compared with the LPS-challenged group (LPS-1.44 ± 0.17-fold vs. HA 1.03 ± 0.09, *p* < 0.01) (Figure 6E). Furthermore, LPS injection significantly increased the number of CD68^+^ phagocytes in the CA3 hippocampal region compared with the control group. Compared with the septic group, the pretreatment of HA reduced the infiltration of CD68^+^ phagocytes (Figure S7).

### 2.6. HA Improved Pulmonary Histological Changes during Endotoxemia

**Figure 7:**
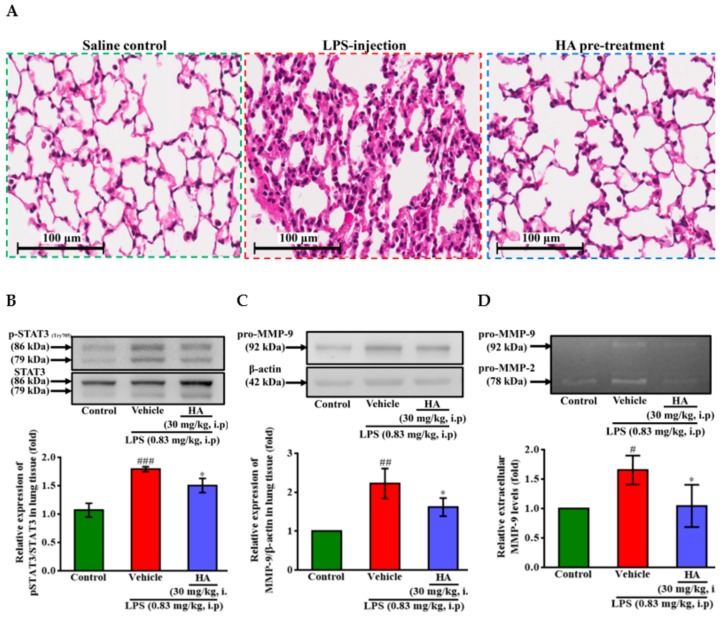
Effect of HA on LPS-mediated histopathological changes and the production of phospho-STAT3 and MMP-9 in lung tissues and BALF. Mice in the different groups administrated with sterile saline or LPS (0.83 mg/kg, i.p) which were sacrificed after behavior tests, and then BALF and lung tissues were collected immediately for following experiments. (**A**) Representative hematoxylin and eosin (H&E)-stained sections of right lung lobes at 400 × original magnification showed exaggerated acute inflammatory infiltration, alveoli destruction and interstitial edema in LPS group (scale bar = 100 µm). Homogenates of the left lung lobes were obtained and analyzed for the expression of phospho-STAT3 (**B**) and pro-MMP-9 (C, *n* = 3) by Western blotting, and assayed for MMPs activity in BALF by gelatin zymography (D, *n* = 3). Data represent group means ± S.D., *n* = 3 to 7 mice/group. ^#^ *p* < 0.05, ^##^ *p* < 0.01 and ^###^ *p* < 0.001 as compared with the saline control group (Control); * *p* < 0.05 as compared with the co-solvent group (vehicle).

Pulmonary edema caused by Gram-negative bacterial infection is the primary manifestation of organ failure and the most common cause of sepsis-related death [29]. It has been reported [30] that STAT3 blocker inhibits the LPS-mediated inflammatory response in mice with acute lung injuries (ALI). One day after intraperitoneal injection of LPS (0.83 mg/kg)*,* histology image analysis was done to assess the effect of HA pretreatment on endotoxemia-mediated lung injury. As shown in Figure 7A, LPS induced pathologic lung injury, including interstitial tissue edema, infiltration of inflammatory cells, and alveolar collapse, which were reduced in the HA-pretreated group compared to the LPS-challenged group. Furthermore, LPS significantly augmented the expression levels of phosphorylated STAT3 (Tyr705) in lung tissue compared to the control group, and HA pretreatment reversed the upregulation of STAT3 phosphorylation induced by LPS (Figure 7B). On the other hand, one day after LPS was challenged, the protein levels of MMP-9 were significantly increased, and pretreatment with HA significantly attenuated the MMP-9 expression induced by LPS of the lung tissues (Figure 7C). Notably, BALF collected from mice injected at one day after LPS treatment also showed an increase in the MMP-9-mediated gelatinolysis by 1.65 ± 0.25 fold compared to control mice. The pretreatment of HA significantly reduced MMP-9 activity in BALF collected from the HA group compared with the LPS-challenged group (LPS-1.65 ± 0.25-fold vs. HA-1.04 ± 0.36 fold, *p* < 0.05) (Figure 7D).

## 3. Discussion

The course of sepsis is often accompanied by many serious complications, such as acute lung injury or sepsis-associated encephalomyelitis, which causes high mortality and poor prognosis [7,8,9]. Systemic inflammatory response during sepsis promotes the abnormal activation of immune cells such as monocyte, which plays a crucial role in organ damage. The infiltration of monocytes causes organ damage, which are associated with the elevation of MMPs and cytokines. MMPs degrade the extracellular matrix leading to abnormal leakage of endothelial tight junctions, which is responsible for the development of edema and organ dysfunction [31,32,33,34]. It had been reported that MMP-9 blockade attenuates the cellular transmigration of monocytes induced by LPS [35]. MMP-2 and MMP-9 blocker could reverse the increase of permeability of the blood-brain barrier and improve acute cognitive alteration associated with sepsis [23]. LPS activates TLR4 downstream signaling including ERK, p38, and NF-κB pathway to upregulate the production of MMP-9 and pro-inflammatory cytokines [26,36]. In the present study, HA pretreatment inhibited LPS-induced ERK1/2 phosphorylation and IκB degradation without cytotoxicity. Notably, HA significantly attenuated LPS-induced MMP-9 mRNA and protein expression and suppressed LPS-mediated extracellular TNF-α, IL-1β, and IL-6 levels. Taken together, these results suggested that HA downregulated THP-1 cells expressing MMP-9 and pro-inflammatory cytokines. These results support our hypothesis that HA improved monocyte-mediated lung injury and anxiety-like behavior during endotoxemia.

In the present study, we showed that HA pretreatment significantly inhibited STAT3 tyrosine phosphorylation in THP-1 cells during LPS stimulation for 24 h. Variety of different stimuli including LPS and pro-inflammatory cytokines activate JAK-STAT3 signaling which exhibits crosstalk between different pathways and involves in the up-regulation of pro-inflammatory mediators, such as IL-6 [25,27,37]. JAK2-STAT3 cascade is activated after 30 min of LPS stimulation, whereas JAK2 blockade strongly inhibits LPS-induced STAT3 (tyr705) phosphorylation [25], and JAK2 also modulates the phosphorylation of the IκBα in neurons [38]. Our study showed that AG490 significantly suppressed both LPS-mediated IκBα degradation and MMP-9 production, indicating that JAK2 was also involved in the regulation of MMP-9 expression during LPS stimulation. Interestingly, the effects of HA on attenuating LPS-induced IκBα degradation and MMP-9 production were more potent than JAK2 inhibitor AG490. Contrary to other studies, we found significant STAT3 tyrosine phosphorylation after LPS challenged THP-1 cells were given LPS for 3 h. Inhibition of ERK-AP-1 pathway downregulate LPS-induced phosphorylation of STAT3 [27]. Similarly, in the present study, pretreatment with MEK inhibitor PD98059 significantly prevented LPS-induced ERK1/2 phosphorylation in THP-1 cells. But, at the same concentration, HA pretreatment was more potent than MEK inhibitors to suppress LPS-mediated STAT3 phosphorylation. These results suggested that HA might suppress phosphorylation of STAT3 via regulating upstream signal molecules, such as JAK2 and Myd88. However, the detailed mechanisms of HA regulating STAT3 activation need further investigation in future studies.

During central nervous system (CNS) inflammation, infiltrated immune cells released chemokines and cytokines that recruited and activated more leukocytes into inflamed site resulting complex long-term immune responses and accumulation of neurotoxic products [39,40]. It has been reported that circulating IL-6 and TNF-α levels were significantly linked to patients with the severity of anxiety, depression and cognitive impairment [10,41]. Similarly, elevated systemic pro-inflammatory cytokines levels were associated with sickness behavior, including locomotor activity deficiency, anxiety- and depressive-like behavior in systemic LPS-challenged mice [42,43,44,45,46]. LPS-induced acute-sickness behavior, including anxiety- and depressive-like behavior was prevented by attenuating among TNF-α, IL-1β, and IL-6 levels of brain and plasma of mice [46,47]. In the present study, intraperitoneal administration of LPS-induced significant locomotor deficits, body weight loss, and anxiety-like behavior, while pretreatment with HA was effective in preventing acute sickness behavior caused by LPS. Additionally, the hippocampus in mice sacrificed after 24 h of behavioral testing showed that pretreatment with HA reduced the level of MMP-9 induced by LPS. It had been indicated that inhibition of MMP-9 and MMP-2 levels could effectively reduce the level of inflammatory cytokines in the brain and improve cognitive impairment caused by sepsis [23]. CCR2^+^/CD68^+^ activated microglia/macrophages were increased following LPS challenge in the hippocampus [48]. Similarly, LPS challenge induced CD68+ inflammatory cell recruitment in the CA3 hippocampal region, and HA pretreatment prevented the infiltration of CD68^+^ microglia/macrophages in the hippocampus induced by LPS injection. However, whether HA could regulate the elevation of pro-inflammatory cytokine levels in the hippocampus and serum in the acute phase (2 h) remains to be confirmed.

During endotoxemia, high levels of TNF-α and IL-6 could activate JAK2-STAT3 cascade in CNS [23,37]. STAT3 was associated with the expression of serotonin reuptake transporter (SERT) known to affect food intake and body weight [49]. The blockade of STAT3 activation in the hippocampus and in the hypothalamus attenuated depression-like behavior and anorexia in mice suffered neuroinflammation, respectively [50,51]. In the present study, intraperitoneal administration of LPS did significantly induce increase STAT3 phosphorylation in the hippocampus, whereas pretreatment with HA significantly lessened the level of phosphorylated STAT3 in the hippocampus, which may help further explain that HA could improve the body weight loss and sickness behavior caused by LPS. However, whether HA could regulate the performance of SERT or serotonin in the hippocampus still needs further verification. On the other hand, STAT3 rapidly activates in the lungs during LPS-induced systemic inflammation, and STAT3 activation occurs before significant lung injury [30,52]. Blockade of STAT3 activity could attenuate LPS-mediated inflammatory cell infiltration and damage in the lung [30]. Our results demonstrated that HA obviously attenuated lung injury features accompanied by suppression of LPS-induced STAT3 activation and MMP-9 production in lung tissues or BALF. These results provided evidence that HA reduced LPS-mediated upregulation of pro-inflammatory mediators and increased vascular permeability.

Although hirsutanol A was reported to be effective on anti-tumor growth in vivo [53], we found HA attenuated the activation of monocyte and microglia [24]. The disruption of blood-brain barrier (BBB) was induced by LPS-associated leukocyte activation. Inhibition of pro-inflammatory TLR4 activation represents an impairment of BBB dysfunction and neuroinflammation [54]. The lung inflammation and injuries are also implicated with monocyte activation [55]. According to these findings, we supposed HA regulated monocyte- and microglia-mediated injuries in brain and lung during inflammatory cascade. However, this study still has some potential limitations. CD11b^high^ GR-1^high^ neutrophils are not only the main source of MMP-9, but also the recruitment of neutrophils play important roles in MMP-9-associated tissue damages during endotoxemia [18]. HA may also block the recruitment of neutrophils, thereby preventing MMP-9-induced lung injuries. However, it is necessary to further investigate the effects of HA on neutrophils during endotoxemia. In addition, according to our findings, HA pre-treatment attenuated STAT-3 phosphorylation induced by LPS challenge. However, we had not directly assessed HA regulates the expression of MMP-9 in hippocampus and lung tissue through regulating STAT3. Clinical sepsis treatment is often administered after the onset of the disease; although HA treatment improves several symptoms induced by endotoxemia in in vivo model, it is still different from the actual clinical situation. In further study, the detail molecular mechanism of HA interacted with STAT3 and the suitability for septic preclinical studies should be determined.

## 4. Materials and Methods

### 4.1. Materials

Hirsutanol A (HA), was obtained from Professor Tzong-Huei Lee [24]. Anti-mouse immunoglobulin (Ig) G-conjugated horseradish peroxidase (HRP) complexes (cat. No NA934) and anti-rabbit IgG-conjugated HRP complexes (cat. No NA931) were purchased from Amersham Biosciences (Sunnyvale, CA, USA). Anti-rat IgG-conjugated CF^TM^488A (cat. No #20014) were purchased from Biotium (Fremont, CA, USA). The rabbit polyclonal antibody (pAb) specific for human native 92-kDa MMP-9 (cat. No AB19016) was purchased from MilliporeSigma (St. Louis, MO, USA). The mouse mAb specific for α-tubulin (cat. No MS-581-P1) was purchased from LabVision/NeoMarkers (Fremont, CA, USA). The rabbit mAb specific for STAT3 (cat. No 12640S), phospho-STAT3 (Tyr705; cat. No 9145S), IκB-α (cat. No 4812S), and p-IKKα/β (Ser176/Ser177) (cat. No 2697S) were purchased from Cell Signaling (Danvers, MA, USA). The rabbit pAb specific for phospho-ERK (Thr202/Tyr204) (cat. No 9101S) and phospho-p38 MAPK (Thr180/Tyr182; cat. No 9211S) were purchased from Cell Signaling (Danvers, MA, USA). The mouse mAb specific for ERK (cat. No 9107S) was purchased from Cell Signaling (Danvers, MA, USA). The rabbit pAb specific for p38 MAPK (cat. No GTX110720), β-actin (cat. No GTX109239) and IKKα (cat. No GTX132964) were purchased from GeneTex (Irvine, CA, USA). Thiazolyl blue tetrazolium bromide (MTT; cat. No M5655-1G), 4-(2-hydroxyethyl)-1-piperazineethanesulphonic acid (HEPES; cat. No H4034-1KG), sodium dodecyl sulfate (SDS; cat. No L3771-500G), phenylmethylsulfonyl fluoride (PMSF; cat. No P-7626), leupeptin (cat. No L2023-5MG), aprotinin (cat. No A1153-5MG), sodium fluoride, sodium orthovanadate (cat. No S6508-10G), sodium pyrophosphate (cat. NO S6422-100G), diethyl pyrocarbonate (DEPC; cat. No 159220-5G), lipopolysaccharide (LPS; cat. No L3880-100mg), parthenolide (PTL; cat. No P0667-5MG), SB203580 (SB; cat. No S8307-1MG), PD98059 (PD; cat. No P215-1MG), AG490 (cat. No 658401-5MGCN) and bovine serum albumin (BSA; cat. No A7906-100G) were purchased from MilliporeSigma (St. Louis, MO, USA). All other chemicals used in this study were of reagent grade.

### 4.2. Cell Cultivation

THP-1 cells (human acute monocytic leukemia line) were obtained from American Type Culture Collection (Manassas, VA, USA). Cells (passage 6 to passage 30) were maintained in RPMI 1640 medium supplemented with 10% heat-inactivated fetal bovine serum (FBS). HEPES (18 mM), NaHCO_3_ (23.57 mM), L-glutamine (3.65 mM), penicillin (90 units/mL) and streptomycin (90 μg/mL) (cat. NO 10378-016, PSQ, GIBCO^TM^, Thermo Fisher Scientific; Waltham, MA, USA). Cells were sub-cultured regularly twice a week (1.2 × 10^6^ cells/ml in 75T flask), as previously described [56].

### 4.3. Gelatin and Reverse Zymography

THP-1 cells (5 × 10^5^ cells/0.5 mL), seeding into 24-well plate in RPMI 1640 medium containing 0.5% FBS were pretreated with or without HA for 15 min and were subsequently stimulated with LPS (50 ng/mL) for 24 h, followed by the collection of the supernatants. The extracellular MMPs levels were evaluated by MMP-mediated gelatinolysis via gelatin zymography. TIMP-1 activity was determined via reverse zymography, as previously described [56,57].

### 4.4. Cellular Viability Assay

THP-1 cells (5 × 10^5^ cells/0.5 mL) were seeded into 24-well plate in RPMI 1640 medium containing 0.5% FBS, then incubated in 24-well plates with indicated concentrations of HA at 37 °C for 24 h. The cytotoxic effects of HA on the viability of THP-1 cells were determined by the MTT colorimetric assay, as previously described [56].

### 4.5. Western Blot Analyses

THP-1 cells (1.0 × 10^6^ cells/mL) were seeded into 6-well plate in RPMI 1640/FBS (0.5%) medium were pretreated with or without indicated concentrations of HA for 15 min, followed by stimulation of LPS (50 ng/mL) for the indicated time. Cells were harvested and lysed as previously described [56]. The samples separated by SDS-PAGE and were blotted onto PVDF or nitrocellulose membranes (cat. NO RPN303F and RPN303D, Hybond™, Amersham™, GE Healthcare, Chicago, IL, USA). The blots were probed with the indicated antibodies and then were visualized by using the enhanced chemiluminescence (cat. NO 32106, Pierce^TM^, Thermo Fisher Scientific; Waltham, MA, USA) and exposure to UVP GelDoc-It2 310 Imaging System (Labrepco; Horsham, PA, USA). The images were cropped, formatted, and adjusted for brightness in Adobe Illustrator. The respective fold was analyzed as previously described [56].

### 4.6. Reverse Transcription Polymerase Chain Reaction (RT-PCR) Analysis

THP-1 cells (1.0 × 10^6^ cells/mL) were seeded into 6-well plate in RPMI 1640/FBS (0.5%) medium were pretreated with or without indicated concentrations of HA for 15 min, followed by stimulation of LPS (50 ng/mL) for 8 h. Total RNA was isolated from THP-1 cells using TRIsure™ (cat. NO BIO-38033, Bioline, Trento, Italy) following the manufacturer’s instructions, and 1 µg of total RNA was reverse transcribed into cDNA using a commercial kit (cat. NO 10928-042, Super Script On-Step RT-PCR system, GIBCO^TM^, Thermo Fisher Scientific; Waltham, MA, USA). The nucleotide sequences of the primers used for amplification were as follows: for MMP-9, sense 5′-CGTGG AGAGT CGAAA TCTCT G-3′ and antisense 5′-CCAAA CTGGA TGACG ATGTC T-3′; for GAPDH, sense 5′-CCACC CATGG CAAAT TCCAT GGCA-3′ and antisense 5′-TCTAG ACGGC AGGTG CAAAT CACC-3′. PCR was performed using the following conditions: 28 cycles of a 15 s denaturation step at 94 °C, a 30 s annealing step at 54 °C, and a 60 s extension step at 72 °C for MMP-9; followed by 25 cycles of a 15 s denaturation step at 94 °C, a 30 s annealing step at 67 °C, and a 60 s extension step at 72 °C for GAPDH. The respective amplified PCR products were analyzed as previously described [56].

### 4.7. Measurement of Cytokine Levels by the Enzyme-Linked Immunosorbent Assay (ELISA)

THP-1 cells (5 × 10^5^ cells/0.5 mL) were seeded into 24-well plate in RPMI 1640 medium (cat. NO 31800-022, GIBCO^TM^, Thermo Fisher Scientific; Waltham, MA, USA) containing 0.5% FBS, then pretreated with or without HA for 15 min and were subsequently stimulated with LPS (50 ng/mL) for the indicated time, and followed by collection of the supernatants. The extracellular levels of TNF-α, IL-6, and IL-1β produced by THP-1 cells were determined using the ELISA Ready-SET-Go! kit (cat. NO #88-7346-88 TNF-α, #88-7066-2 IL-6, and #88-7010-88 IL-1β, eBioscience; San Diego, CA, USA). The quantitative levels of the cytokines were analyzed as previously described [58].

### 4.8. LPS-Induced Endotoxemia In Vivo and Drug Treatment

Male C57BL/6 mice (8–10 weeks, 25–28 g) were purchased from bioLASCO, Taiwan Co., Ltd (Taipei, Taiwan). The Institutional Animal Care and Use Committee (IACUC) of Taipei Medical University approved the animal experiments in this study (LAC-2015-0195). To develop mouse endotoxemia models, LPS (E. coli LPS, serotype 0127:B8) was dissolved in pyrogen-free sterile saline (0.9% *w*/*v* NaCl) at a concentration of 0.5 mg/mL and intraperitoneally injected at the dose of 0.83 mg/kg of body weight [45]. C57BL/6 mice were assigned to receive a volume of 1.66 mL/kg LPS or an equal amount of normal saline via intraperitoneal injection. For evaluating the effects of HA on the endotoxemia-mediated sickness behavior, anxiety-like behavior and lung inflammation, HA was dissolved in co-solvent as cremophor EL (cat. NO C5135-500G, MilliporeSigma, St. Louis, MO, USA)/ethanol (1:1) and was diluted (1:10) with pyrogen-free sterile saline (0.9% *w*/*v* NaCl), and intraperitoneally administered 30 min before LPS-injection at 30 mg/kg of body weight. Animals were randomized into three experimental groups (*n* = 7) for behavioral and biochemical assessment. The group III (HA group) were intraperitoneally administrated with HA, and the group I (Saline control group) group II were injected with an equal volume of vehicle (co-solvent) before LPS injection. After 30 min, the group I challenged with Saline, group II and group III challenged with LPS (0.83 mg/kg, i.p.), respectively. LPS-mediated behavior impairments were assessed by OFT and EPM 2/24 h and 25 h, respectively, post-LPS or saline challenge. Animals were continuously monitored for 26 h. At the end of the above behavioral test, the mice were sacrificed and the target organs were harvested.

### 4.9. Behavioral Testing

The Open Field Test (OFT)—The OFT was performed in an opaque white plastic box (46.5 × 50.5 × 50.5 cm) and illuminated from overhead (an average of 30 lux). The central zone was defined as the 23.25 × 25.25 cm interior portion of the box. Mice were gently and individually placed in the center of the apparatus and allowed to explore for 5 min. The test period was recorded by a video camera located above the center of the apparatus. Between trials, the box was cleaned with 75% ethanol. Exploratory locomotor activity (eLMA) was used to reflect acute sickness behavior and was measured as the total distance traveled by each mouse in the box. The anxiety-like phenotype of an individual mouse’s behavior was assessed by measuring the percentage of time spent in the central or peripheral area of the box [45]. All of the variables were analyzed using Ethovision XT 11 tracking software (Noldus, Leesburg, VA, USA).

The Elevated Zero Maze test (EZM test)—The maze was constructed of opaque white plastic in a circular track 5 cm wide, 68 cm in diameter, elevated 51 cm from the floor, and illuminated from overhead (average 30 lux). The maze was divided into four quadrants of equal length with two opposing open quadrants and two opposing closed quadrants with white plastic walls 12 cm in height. Mice were gently placed in the center in the center of a closed quadrant. An overhead video camera recorded the location of the mice in a 5 min test period. The anxiety-like behavior was assessed by measuring the percent time and entries in the open quadrants. The maze was cleaned with 75% ethanol between mice. All of the variables were analyzed using Ethovision XT 11 tracking software (Noldus, Leesburg, VA, USA).

### 4.10. Bronchoalveolar Lavage Fluid (BALF) and Lung Tissues Collection

At the end of the above behavioral test, the mice were sacrificed by an excess of anesthetic. The chest was opened, a 20 Gauge blunt needle was inserted, and 0.8 mL of ice-cold sterile PBS was dropped into the lungs through a tracheal incision. The recovered BALF was centrifuged at 500× *g* for 10 min, the protein levels of the cell-free supernatant were quantified and used to evaluate the pro-MMP-9 concentration by gelatin zymography [59]. The lung tissues were harvested immediately after collection of BALF. Right lobes of the lung were fixed in 4% formalin and paraffin embedded and stained with hematoxylin and eosin (HE). Whole digital images were obtained using virtual microscopy (Aperio CS2 Leica Microsystems, Wetzlar, Germany). Tagged Image File Format (TIFF) images extracted using image processing software (ImageScope, ver. 9.1.19.1567, Leica Microsystems). Left lobes of lung were immediately frozen in liquid nitrogen, and 50 mg samples of frozen lung tissue were subsequently homogenized with 2 mL Precellys homogenization tube containing three 1.4-mm and three 2.8-mm ceramic beads (Bertin Technologies, Rockville, Maryland) with 7 volumes (7 mL/g tissue) of homogenization buffer which containing 1× protease inhibitor (cat. NO 11 873, 580 001, Roche, Mannheim, Germany). The samples were homogenized (Minilys^®^; Bertin Technologies, Rockville, Maryland) twice at 5000 rpm for 30 s and then centrifuged at 5000× *g* for 5 min at 4 °C. The collected supernatant was incubated at 4 °C for 30 min, then centrifuged at 12,000× *g* for 20 min and then stored at −80 ° C before additional experiments.

### 4.11. Hippocampus Tissues Collection

The hippocampus tissue was harvested immediately after the lung tissues were preserved. The hippocampus was quickly removed on an ice-cold metal plate, and was transferred into a 2 mL Precellys homogenization tube containing three 1.4-mm ceramic beads (Bertin Technologies, Rockville, MD, USA) with a homogenization buffer (7 mL/g tissue) which containing 1× protease inhibitor (cat. NO 11 873, 580 001, Roche, Mannheim, Germany). The samples were homogenized (Minilys^®^; Bertin Technologies, Rockville, MD, USA) twice at 5000 rpm for 15 s and then centrifuged at 5000× *g* for 5 min at 4 °C. The collected supernatant was incubated at 4 °C for 30 min, then centrifuged at 12,000× *g* for 20 min and then stored at −80 °C before additional experiments.

### 4.12. Statistical Analyses

The experimental results are represented as the means ± S.D. for the number of observations. Analysis of experiments was performed using one-way analysis of variance (ANOVA) followed by a multiple comparison test (Student–Newman–Keuls test) via SigmaStat v3.5 software (SYSTAT Software, San Jose, CA, USA). *p* < 0.05 was considered to indicate statistical significance.

## 5. Conclusions

These results indicated that HA strongly attenuated MMP-9, TNF-α, and IL-6 via regulating the TLR4 downstream signaling pathways, including ERK-1/2, NF-κB, and STAT3. Our findings demonstrated that the novel fungal sesquiterpene HA could provide new opportunities for the development of different strategies on pro-inflammatory cytokines- and MMP-9-associated diseases, such as endotoxemia.

## Supplementary Materials

Supplementary Materials are available at https://www.mdpi.com/1660-3397/17/6/360/s1. Supplementary Method-Immunohistochemistry. Figure S1. Effect of HA on monocytic MMP-9-mediated gelatinolysis; Figure S2. Effect of HA on the pro-inflammatory cytokines production of THP-1 cells; Figure S3. Effect of the combination of LPS and HA on the cell viability of THP-1 cells; Figure S4. Effect of HA on LPS-mediated phosphorylation of IKKα in THP-1 cells; Figure S5. Effect of HA on the STAT3 and NF-κB cascade in THP-1 cells; Figure S6. Effect of HA on LPS-induced body weight loss and hypothermia; Figure S7. Effect of HA the numbers of CD68^+^ inflammatory cells in the hippocampus following systemic LPS challenge.
