# Supplementary material for: Hirsutanol A Attenuates Lipopolysaccharide-Mediated Matrix Metalloproteinase 9 Expression and Cytokines Production and Improves Endotoxemia-Induced Acute Sickness Behavior and Acute Lung Injury
Source: Mar Drugs. 2019 Jun 17;17(6):360. doi: 10.3390/md17060360 (PMC6627105; doi:10.3390/md17060360)
Supplement: Supplementary file 1 [file marinedrugs-17-00360-s001.pdf]

# Hirsutanol A Attenuates Lipopolysaccharide-Mediated Matrix Metalloproteinase 9 Expression and Cytokines Production and Improves Endotoxemia-Induced Acute Sickness Behavior and Acute Lung Injury

**Jing-Shiun Jan [1,2], Chih-Hao Yang [1,2], Mong-Heng Wang [3], Fan-Li Lin [1,2], Jing-Lun Yen [1,2], Irene Hsieh [1,2],Maxim Khotimchenko [4], Tzong-Huei Lee [5,†] and George Hsiao [1,2,6,†,*]**

[1] Graduate Institute of Medical Sciences, College of Medicine, Taipei Medical University, Taipei 110, Taiwan; d119101004@tmu.edu.tw (J.-S.J.); chyang@tmu.edu.tw (C.-H.Y.); fllin@tmu.edu.tw (F,-L.L.); m120102039@tmu.edu.tw (J.-L.Y.); m120105021@tmu.edu.tw (I.H.)

[2] Department of Pharmacology, School of Medicine, College of Medicine, Taipei Medical University, Taiwan

[3] Department of Physiology, Augusta University, Augusta, GA, USA; mwang@augusta.edu (M.-H.W.)

[4] School of Biomedicine, Far Eastern Federal University, Vladivostok 690091, Russian Federation; khotimchenko.my@dvfu.ru (M.K.)

[5] Institute of Fisheries Science, National Taiwan University, Taipei 106, Taiwan; thlee1@ntu.edu.tw (T.-H.L.)

[6] Ph.D. Program in Biotechnology Research and Development, College of Pharmacy, Taipei Medical University, Taipei 110, Taiwan

* Correspondence: geohsiao@tmu.edu.tw (G.H.); Tel.: +886-2-2737-4622 (G.H.); Fax: +886-2-2737-4622 (G.H.)

† These authors contributed equally to this work.

## Supplementary Method

*Immunohistochemistry*

C57BL/6 mice were assigned to receive a volume of 1.66 mL/kg LPS or an equal amount of normal saline via intraperitoneal injection. For evaluating the effects of HA on the endotoxemia-mediated sickness behavior, anxiety-like behavior and lung inflammation, HA was dissolved in co-solvent as cremophor EL/ethanol (1:1) and was diluted (1:10) with pyrogen-free sterile saline (0.9% w/v NaCl), and intraperitoneally administered 30 min before LPS-injection at 30 mg/kg of body weight. Animals were randomized into three experimental groups (n=3) for behavioral and biochemical assessment. The group III (HA group) were intraperitoneally administrated with HA, and the group I (Saline control group) group II were injected with an equal volume of vehicle (co-solvent) before LPS injection. At 24 h post-LPS or saline challenge, animals were deeply anesthetized with isoflurane and perfused transcardially with PBS, followed by filtered perfusion solution (4% paraformaldehyde and 0.5% picric acid). After the perfusion, the brain was removed and postfixed in the same perfusion solution overnight. Following fixation, whole brains were cryoprotected in filtered PBS containing 30% sucrose over 2 days. Brain sections (35 μm) were cut in a cryostat and processed for immunofluorescence. All histological data were obtained from sections taken from coronal planes, and the center of the field of view was focused on the hippocampal region. For double immunofluorescence, the sections were first blocked with PBS containing 6% goat serum and 0.25% Triton X-100 for 1 h at room temperature. After washing, the sections were incubated overnight at 4°C with PBS containing 3% normal goat serum, 0.1% Triton X-100, and mixture of primary antibodies: CCR2 antibody (rabbit, 1:3000) and phosphor-STAT3 antibody (mouse, 1:3000); CCR2 antibody and MMP-9 antibody (mouse, 1:3000); CCR2 and CD68 antibody (rat, 1:3000) diluted with SignalPlus antibody enhancer solution 1 (GeneTex, Irvine, CA, USA), respectively. The sections were then incubated overnight at 4°C with a mixture of CFTM594-conjugated (1:3000), CFTM488A-conjugated (1:3000) or FITC-conjugated secondary antibodies (1:3000) diluted with SignalPlus enhancer solution 2 (GeneTex, Irvine, CA, USA). The slices were imaged using a fluorescence microscope (EVOS® FL Cell Imaging System, Thermo Scientific, Waltham, MA, USA).

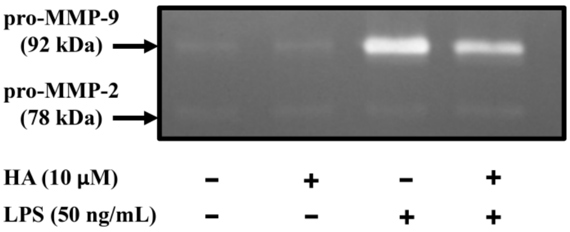


**Figure S1. Effect of HA on monocytic MMP-9-mediated gelatinolysis.** THP-1 cells ($5 \times 10^5$ cells/0.5 mL) were dispensed onto 24-well plates and treated with or without LPS (50 ng/mL) for 24 h. Cells were treated with HA (10 µM) or vehicle for 15 min before LPS treatment. Cell-free supernatants were then assayed for MMPs by gelatin zymography.

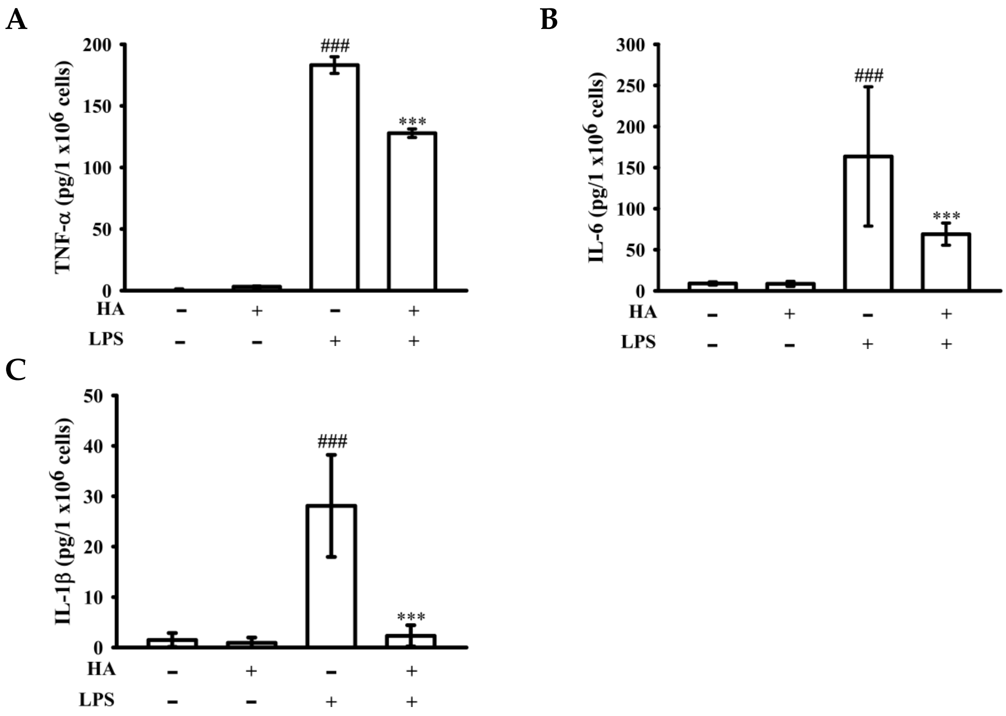


**Figure S2. Effect of HA on the pro-inflammatory cytokines production of THP-1 cells.** THP-1 cells (5 × $10^5$ cells/0.5 mL) were dispensed onto 24-well plates and were treated with HA (5 μM) or vehicle for 15 min followed by treatment without or with LPS (50 ng/mL) for 4 h (A) and 24 h (B, C). Cell-free supernatants were then assayed for the level of TNF-*α* (A), IL-6 (B) and IL-1β (C) by ELISA. Data represent means ± S.D. from three independent experiments. $^{\#\#\#}P<0.001$ as compared with the resting; $^{***}P<0.001$ as compared with the vehicle followed by treatment with LPS.

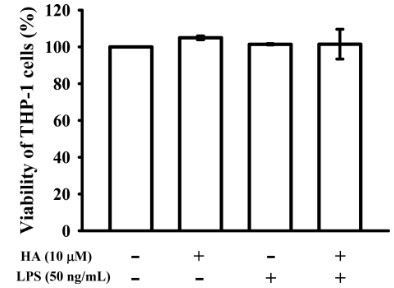


**Figure S3. Effect of the combination of LPS and HA on the cell viability of THP-1 cells.** THP-1 cells (5 × $10^5$ cells/0.5 mL) were dispensed onto 24-well plates and were treated with HA (10 μM) or vehicle for 15 min followed by treatment without or with LPS (50 ng/mL) for 24 h. Cell viability was quantified by the ability of mitochondria to reduce the tetrazolium dye MTT in viable cells. Data represent means ± S.D. from three independent experiments.

\

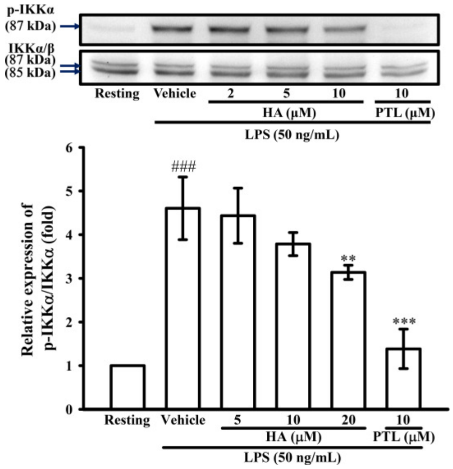


**Figure S4. Effect of HA on LPS-mediated phosphorylation of IKK$\alpha$ in THP-1 cells.** THP-1 cells ($10^6$ cells/mL) were dispensed onto 6-well plates and treated with the indicated concentrations of HA (2, 5 and 10 μM), parthenolide (PTL, 10 μM) or vehicle followed by treatment of LPS (50 ng/mL) for 60 min. Cell lysates were obtained and analyzed for the phosphorylation of IKK$\alpha$ by Western blotting. Data represent means ± S.D. from three independent experiments. $^{###}P<0.001$ as compared with the resting; $^{**}P<0.01$ and $^{***}P<0.001$ as compared with the vehicle.

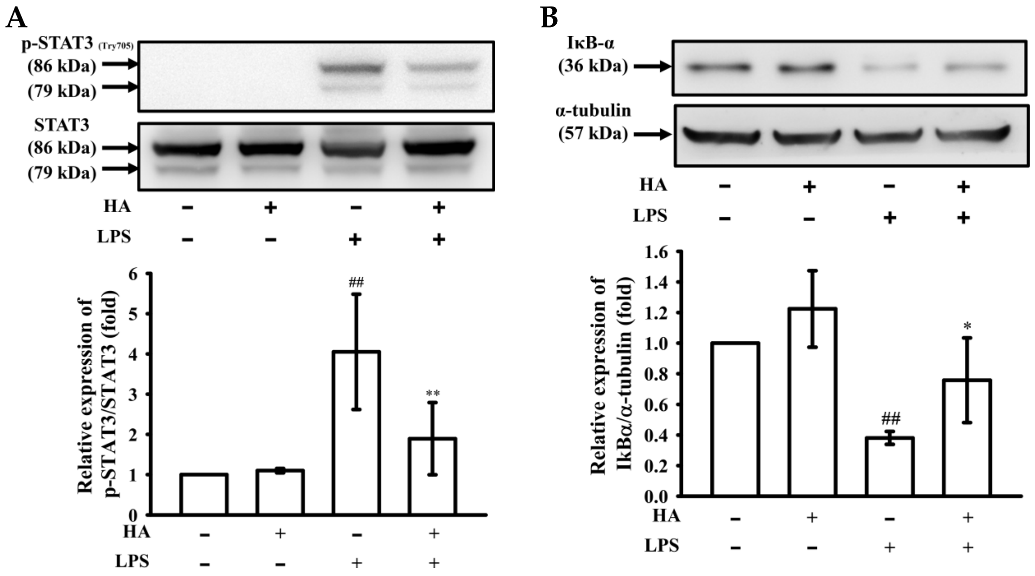


**Figure S5. Effect of HA on the STAT3 and NF-κB cascade in THP-1 cells.** THP-1 cells ($10^6$ cells/mL) were dispensed onto 6-well plates and were treated with HA (10 μM) or vehicle for 15 min followed by treatment without or with LPS (50 ng/mL) for 24 h (A), and 60 min (B), respectively. Cell lysates were obtained and analyzed for the level of phosphorylated-STAT3 and IκB$\alpha$ degradation by Western blotting. Data represent means ± S.D. from three independent experiments. $^{\#\#}P<0.01$ as compared with the resting; $^{*}P<0.05$ and $^{**}P<0.01$ as compared with the vehicle followed by treatment with LPS.

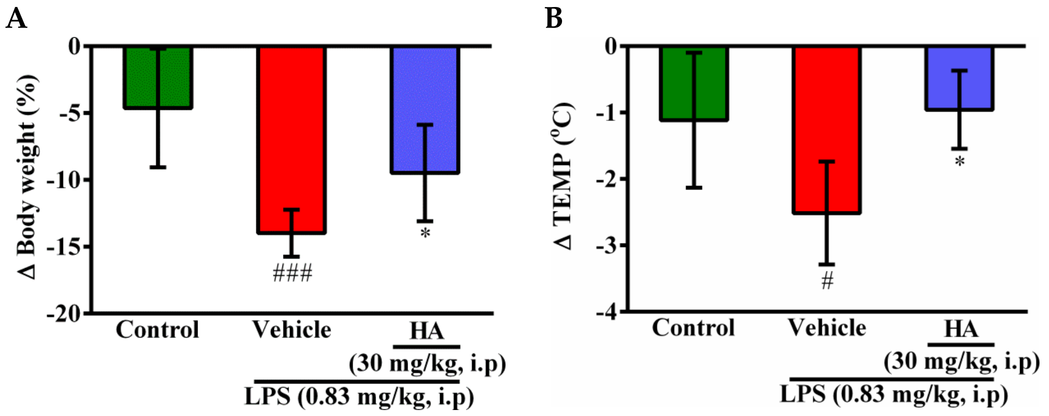


**Figure S6. Effect of HA on LPS-induced body weight loss and hypothermia.** (A) Percentage of body weight changes (ΔBody weight %) measured 24 h after administration with LPS (0.83 mg/kg, i.p) or sterile saline which divided by 0 h body weight and then multiplied by 100 %. (B) Mice were injected with LPS (0.83 mg/kg, i.p) or sterile saline was then tested for body temperature changes (ΔTEMP℃) which divided by 0 h body temperature and then multiplied by 100 %. Data represent group means ± S.D., n=7 mice/group. $^{\#}P<0.05$ and $^{\#\#\#}P<0.001$ as compared with the saline control group (Control); $^{*}P<0.05$ as compared with the co-solvent group (vehicle).

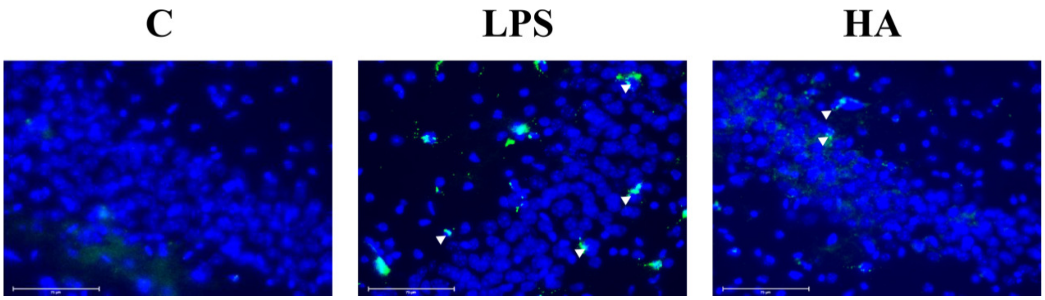


**Figure S7. Effect of HA the numbers of CD68⁺ inflammatory cells in the hippocampus following systemic LPS challenge.** After LPS or saline challenge for 24 h, CD68 and DAPI labeling were colocalized in CA3 hippocampal region of the three group mice were imaged by fluorescence microscopy (Scale bar: 75 μm). The arrowhead highlights CD68+ activated microglia/monocyte.
